# Structure–Function Correlation in Cobalt-Induced Brain Toxicity

**DOI:** 10.3390/cells13211765

**Published:** 2024-10-24

**Authors:** Basel Obied, Stephen Richard, Alon Zahavi, Dror Fixler, Olga Girshevitz, Nitza Goldenberg-Cohen

**Affiliations:** 1 The Krieger Eye Research Laboratory, Bruce and Ruth Faculty of Medicine, Technion—Israel Institute of Technology, Haifa 3525433, Israel; basel.obied01@gmail.com (B.O.); steverit11@gmail.com (S.R.); 2 Department of Ophthalmology and Laboratory of Eye Research, Felsenstein Medical Research Center, Rabin Medical Center, Petach Tikva 4941492, Israel; alonzahavi@gmail.com; 3 Faculty of Medicine, Tel Aviv University, Tel Aviv 69978, Israel; 4 Faculty of Engineering and Institute of Nanotechnology and Advanced Materials, Bar Ilan University, Ramat Gan 5290002, Israel; dror.fixler@biu.ac.il (D.F.); olga.girshevitz@biu.ac.il (O.G.); 5 Department of Ophthalmology, Bnai Zion Medical Center, Haifa 31048, Israel

**Keywords:** cobalt, neurodegeneration, minocycline, Morris water maze, open field test, electron microscopy, behavioral tests, MRI, PIXE

## Abstract

Cobalt toxicity is difficult to detect and therefore often underdiagnosed. The aim of this study was to explore the pathophysiology of cobalt-induced oxidative stress in the brain and its impact on structure and function. Thirty-five wild-type C57B16 mice received intraperitoneal cobalt chloride injections: a single high dose with evaluations at 24, 48, and 72 h (*n* = 5, each) or daily low doses for 28 (*n* = 5) or 56 days (*n* = 15). A part of the 56-day group also received minocycline (*n* = 5), while 10 mice served as controls. Behavioral changes were evaluated, and cobalt levels in tissues were measured with particle-induced X-ray emission. Brain sections underwent magnetic resonance imaging (MRI), electron microscopy, and histological, immunohistochemical, and molecular analyses. High-dose cobalt caused transient illness, whereas chronic daily low-dose administration led to long-term elevations in cobalt levels accompanied by brain inflammation. Significant neurodegeneration was evidenced by demyelination, increased blood–brain barrier permeability, and mitochondrial dysfunction. Treated mice exhibited extended latency periods in the Morris water maze test and heightened anxiety in the open field test. Minocycline partially mitigated brain injury. The observed signs of neurodegeneration were dose- and time-dependent. The neurotoxicity after acute exposure was reversible, but the neurological and functional changes following chronic cobalt administration were not.

## 1. Introduction

Cobalt is an essential trace element in humans. The average daily dietary intake in adults is only 0.13 to 0.48 μg/kg body weight, and the physiologic blood concentrations are below 6 μg/L [1]. Cobalt toxicity is difficult to detect and therefore often underdiagnosed. It may occur following exposure to batteries, hard alloys, reagents, pigments, dyes, and beer additives [1]. Failed metal-on-metal arthroplasty implants have been implicated in chronic systemic cobalt toxicity with related brain, heart, and thyroid dysfunction [2,3,4,5]. Neurological symptoms of cobalt toxicity include tremors, lack of coordination, vertigo, mood disturbances, cognitive decline, hearing loss, and visual impairment [6]. 

The pathophysiology of neurological cobalt toxicity is multifactorial. Several mechanisms have been suggested. In vitro studies detected oxidative stress and inflammation in astrocytes [7], neurons [8,9,10,11,12,13], and glial cells [14,15]. Others found that cobalt induced cell death by both apoptosis and necrosis [16] in addition to DNA fragmentation [17], caspase activation [18], increased production of reactive oxygen species [17], and elevated levels of p53 [19]. Cobalt has also been shown to induce hypoxia through stabilization of the alpha subunits of hypoxia-inducible factor (HIF) [20]. 

In vivo studies of cobalt neurotoxicity are limited and mostly related to cobalt-induced hypoxia and the expression of hypoxia-related biomarkers [21]. Caltana et al. found that direct application of cobalt to the cerebral cortex resulted in ischemic damage [22], and Zheng et al. suggested that cobalt-induced neurodegeneration is a consequence of the inactivation of Pin1, a key regulatory enzyme in neuronal processes [23]. Accordingly, a loss-of-function mutation in *Pin1* has been identified in humans with Alzheimer’s disease [24,25].

Cobalt toxicity has been reported to be associated with an inflammatory component primarily linked to microglial activation [7,14,15]. To address this possible inflammatory response, we utilized minocycline, an anti-inflammatory drug known for its ability to modulate macrophage activity. Minocycline has the theoretical potential to mitigate cobalt-induced inflammation, as it has been shown to reduce hypoxia-induced microglial excitation [26] and provide protection against neonatal hypoxic insults [27].

The aim of this experimental study was to explore the pathophysiology of cobalt-toxicity-induced oxidative stress in the brain and to investigate the correlation between structural damage and functional impairment.

## 2. Materials and Methods

### 2.1. Study Animals

All mice involved in this study were housed and treated following the guidelines outlined by the Association for Research in Vision and Ophthalmology (ARVO) for the Use of Animals in Ophthalmic and Vision Research, as well as the regulations of the National Institutes of Health. The animal protocols received approval from the Animal Research Committee at Rabin Medical Center, Israel (RMC_02062021), and the Technion—Israel Institute of Technology, Israel (IL0790521). The study is reported in accordance with ARRIVE guidelines.

**Figure 1:**
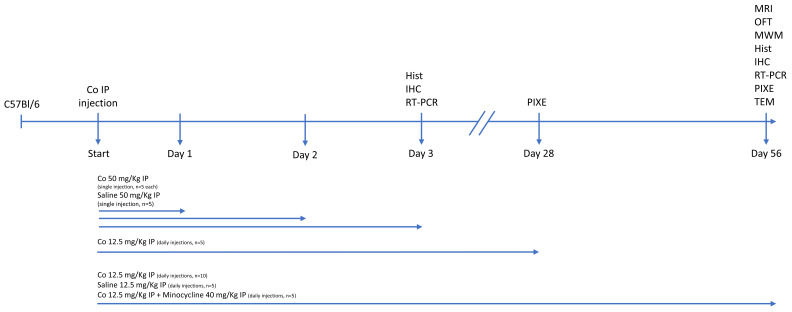
Overview of experimental design for cobalt exposure in mice. Thirty-five wild-type mice were divided into two groups: mice treated with a single high-dose IP injection of CoCl2 observed for 3 days (*n* = 5 each) and mice treated with a daily low-dose IP injection of CoCl2 for 28 (*n* = 5) or 56 days (*n* = 15). An additional 10 mice served as controls. The doses administered and follow-up times and studies performed are noted. Hist—histology, IHC—immunohistochemistry, IP—intraperitoneal, MRI—magnetic resonance imaging, TEM—transmission electron microscopy, OFT—open field test, MWM—Morris water maze, RT-PCR—real-time polymerase chain reaction, PIXE—particle-induced X-ray emission, TEM—transmission electron microscopy.

Cobalt chloride (CoCl_2_; Sigma Aldrich, St. Louis, MO, USA) was administered to 35 C57B16 wild-type mice divided into two groups (Figure 1). Group 1 received a single high-dose intraperitoneal (IP) injection and was observed for 24, 48, or 72 h (*n* = 5, each). Group 2 received a low-dose daily IP injection for 28 (*n* = 5) or 56 days (*n* = 15). Part of the 56-day low-dose subgroup received additional IP treatment with minocycline (M9511, Sigma-Aldrich) (*n* = 5). A third (control) group received a daily IP saline injection for 24 h or 56 days (*n* = 5, each). 

### 2.2. Injection Dosage

CoCl_2_ was dissolved in 100 µL saline solution, and the injection dosage, based on the literature [23], was calculated per kilogram mouse body weight. Thus, a 30 gr mouse in the high-dose group received 50 mg/kg CoCl_2_, and a 30 gr mouse in the low-dose group received 12.5 mg/kg CoCl_2_. The concentration used was 15 mg/mL for the high dose and 3.75 mg/mL for the low dose. Each injection consisted of a total volume of 0.1 mL solution. The minocycline dose was 45 mg/kg, based on the literature [26], with a concentration of 13.5 mg/mL and a volume of 0.1 mL. The control group was injected with 0.1 mL of normal saline (NaCl 0.9%) (Figure 1).

### 2.3. Injection Procedures 

IP injections were performed with a 27-gauge needle inserted at the lower right quadrant of the abdomen at a 30–40º angle to the horizontal plane. The plunger was pulled back to ensure negative pressure to avoid hitting an artery or vein. CoCl_2_ solution, minocycline, or saline was injected at the predetermined concentration per group allocation. 

Cobalt-injected mice were euthanized by carbon dioxide asphyxiation at 24 h, 48 h, 72 h, 28 days, and 56 days. Minocycline-injected mice were euthanized after 56 days, and control mice, after 24 h and 56 days.

Figure 1 shows the points at which the clinical, behavioral, and laboratory evaluations were performed in relation to the time of injection/euthanization. 

### 2.4. Behavioral Tests

#### 2.4.1. Open Field Test

The OFT is used to evaluate exploratory behavior and anxiety in rodents. The apparatus consists of a gray lusterless box (50_L_ × 50_W_ × 40_H_ cm) situated in a dimly lit room (50_L_×). Each mouse was placed in a corner of the open field facing the wall and given 5 min of free exploration. Behavior was recorded and analyzed with ANY-maze software v 7.46 (Stoelting Co., Wood Dale, IL, USA). In order to calculate the center crossing and anxiety index (distance moved in the periphery/total distance moved × 100), the maze was virtually divided into the center and periphery (7 cm width from the walls).

#### 2.4.2. Moris Water Maze

The MWM is used to evaluate spatial learning in rodents. The test is conducted in a circular white plastic tank (1.2 m diameter with a 0.5 m high rim) filled with water to a depth of 30.0 cm. Temperature is maintained at 25 ± 1 °C. The only means of escape from the water is a hidden platform, 10 cm in diameter, lying 1 cm beneath the water surface. The maze itself has no landmarks; the only landmarks are located outside the maze, on the surrounding walls. Mice were randomly placed (facing the wall) at one of four starting positions (N, E, W, and S) and allowed to swim freely for a maximum of 90 s or until reaching the hidden platform. At the end of each trial, each mouse was led to or left on the platform for 10 s.

Spatial acquisition training consisted of four trials per day, with a 2-min inter-trial interval, for 6 days. Two hours after the last trial (day 6), the platform was removed, and a probe trial was conducted wherein mice were placed in the maze for 60 s. Their behavior was recorded and analyzed with ANY-maze software (Stoelting). 

### 2.5. Histology 

Brains were fixed in 4% paraformaldehyde and embedded in paraffin. Brains were cut into coronal sections of 5 μm and mounted on slides which were then stained with hematoxylin and eosin (H&E) for a light microscopy scanner (Pannoramic 250 Flash III, 3DHISTECH, Budapest, Hungary), with three consecutive sections per slide.

### 2.6. Immunohistochemistry

Brains and optic nerves were fixed in 4% paraformaldehyde, placed overnight in 30% sucrose dissolved in phosphate-buffered saline (PBS) (1×; Biological Industries, Beit HaEmek, Israel) at 4 °C, and embedded in optimum cutting temperature compound (Scigen Scientific, Gardena, CA, USA). Brain and optic nerve sections (40 and 15 µm, respectively) were placed on slides and washed with PBS prior to blocking with 5% fetal calf serum and 1% Triton X-100 for 1 h. Brain sections were then incubated with the primary anti-albumin antibody (1:200, ZOTAL Biologicals and Instrumentation, Tel Aviv, Israel), primary anti-IBA-1 (1:500, ZOTAL), primary anti-Neun (1:500, Merck Millipore, Burlington, MA, USA), anti-glial fibrillary acidic protein (GFAP) (1:500, ZOTAL), and anti-VEGF (1:200, ZOTAL) at 4 °C overnight. Optic nerve sections were incubated with anti-MBP (1:400, ZOTAL) at 4 °C overnight. Slides were washed with PBS and incubated with the secondary antibodies, goat anti-rabbit Alexa fluor 647 (diluted 1:1000, ZOTAL) and goat anti-chicken IgG NL-577 (diluted 1:200, R&D Systems-Biotest, Minneapolis, MN, USA), at room temperature for 1 h. Sections were counterstained with 4′,6-diamidino-2-phenylinodole (DAPI) (Molecular Probes Invitrogen, Eugene, OR, USA) to reveal cell nuclei. Images were obtained using a confocal fluorescence microscope (Zeiss LSM700, Munchen, Germany). 

GFAP immunofluorescence intensity was quantified using Fiji ImageJ software (1.54f, National Institute of Health USA). Identical parameters of laser configuration and areas sampled for analysis were ensured. 

Z-stack images of Iba1-positive cells from the brain sections were acquired using a Zeiss confocal microscope (LSM700, Zeiss, Munchen, Germany) with a 20× objective. The imaging parameters and software set-up remained constant for all photomicrograph acquisition in the experiment. Using a mouse brain atlas, we specifically selected sections corresponding to the hippocampus and frontal cortex for analysis. Fiji ImageJ software (1.54f, National Institute of Health) with the Sholl analysis plugin was utilized for manual morphological analysis, as previously described [28,29]. In brief, Z-stacks were imported into the software for analysis. Slices were processed as maximum intensity projections, which were the threshold for creating a binary mask. The circularity index (CI) was calculated by measuring the area (black) and the perimeter (red) of a cell from the binary mask (4π[area]/[perimeter]^2^) [30]. For Sholl analysis, the maximum radius of the cell soma and the radius extending beyond the longest cell branch were determined. The Fiji Sholl analysis plugin was used with its default parameters (enclosing radius cutoff = 1 intersection, radius step size = 1.00 microns, and Sholl method = linear). At least 20 cells were analyzed for each mouse.

Brain longitudinal cross-sections were cut 40 μm thick for in situ TUNEL apoptosis assay (Roche Diagnostics GmbH, Roche Applied Science, Mannheim, Germany); staining was performed with the fluorescein-tagged apoptosis detection system. The results were analyzed with a confocal microscope (Zeiss LSM700) at 488 nm wavelength. 

### 2.7. Molecular Analysis

At 24 and 72 h after the administration of a high-dose cobalt injection, whole brains were harvested and preserved in RNAlater solution (Invitrogen, Life Technologies, Carlsbad, CA, USA) at −80 °C. Total RNA was extracted using TRIzol reagent (Invitrogen, CA, USA) following the manufacturer’s instructions and subsequently reverse-transcribed into cDNA using random hexamers (Bioline, London, UK) and Moloney murine leukemia virus (M-MLV) reverse transcriptase (Promega, Madison, WI, USA).

A two-step real-time quantitative PCR was utilized with the PCR Sequence Detection System (Prism 7900, Applied Biosystems, Foster City, CA, USA) to analyze mRNA expression levels for genes related to oxidative stress, apoptosis, and ischemia, including hypoxia-inducing factor-2 alpha (Hif2α), Bcl-2, Bax, Superoxide dismutase 1 (Sod1), and heme oxygenase 1 (Hmox1). To normalize the cDNA input, mouse actin beta (Actb) was used as a reference gene. The primers used are detailed in Table 1. The reactions were carried out in a 20 μL mixture containing 4 μL of cDNA, 0.5 μM of each forward and reverse primer, and a buffer included in the Master Mix (SYBR Green I; Applied Biosystems, Foster City, CA, USA). Each gene was tested in duplicate to minimize variability, and the average was calculated for each time point. Efficiency corrections for the threshold cycle were applied, and melting curves were generated using cDNA for each gene PCR assay.

### 2.8. Particle-Induced X-Ray Emission (PIXE)

Direct elemental PIXE analysis was used to determine cobalt levels in biological fluids (blood and urine) and tissues (brain, heart, and liver). Dried matrix spots (DMSs) were measured from small sample volumes (50 μL) using internal standard addition. Whatman^TM^ qualitative filter paper, grade 1 (Sigma-Aldrich, Taufkirchen, Germany), was pre-cut into discs 12 mm in diameter for DMS sample preparation, and polystyrene multi-well plates (Thermo Fisher Scientific, Waltham, MA, USA) were used for sample support. Micropipettes (Thermo Fisher Scientific) were used for all volumetric sample handling. The liquid samples were absorbed on the paper substrate. 

Standard working solutions of 1, 2, 5, 10, 20, 50, 100, and 200 mg/L cobalt were prepared in 1% nitric acid using a certified standard solution of 1000 mg/L cobalt (Sigma-Aldrich) to validate sample homogeneity, dynamic range, repeatability/reproducibility, and limit of detection/quantification and to determine the calibration curve (linearity). Standard working solutions and biological fluid samples, each with a volume of 50 μL, were pipetted on filter paper, placed on plastic support, and dried under ambient conditions. Vanadium (20 μg/mL) was used as an internal marker for calibration in all experiments.

For PIXE measurements, a 2.6 MeV proton beam obtained from a 1.7 MV Pelletron Accelerator (National Electrostatics Corporation, Middleton, WI, USA) was collimated to a beam size of 1 mm. The X-ray spectra were collected using a FAST SDD^®^ silicon drift detector (Amptek, Bedford, MA, USA) with a silicon nitride (Si_3_N_4_) window, with an active area of 25 mm^2^, nominal thickness of 0.5 mm, and 128 keV nominal resolution at 5.9 keV. A 12 μm Mylar foil was placed in front of the detector window to filter out the backscattered protons and low-energy X-rays. The target was positioned at 0°, with the detector at 40° to the beam normal. The DMS samples were mounted on the silicon wafer attached to a sample holder with double-sided adhesive carbon tape and placed in the target chamber under high vacuum (7–10 mbarTorr). The energy calibration was performed using brass (70:30 Cu/Zn) and TiNb. Glass certified reference materials (CRMs) NIST 610 and NIST 620 were used for energy calibration and quality control measurements. All samples were irradiated up to the total collected charge of 5 μC. GUPIX software, version 3.0.3 (Guelph, ON, Canada), was used to analyze the collected PIXE spectra.

### 2.9. Electron Microscopy

Brains and optic nerves were dissected, cleaned, and placed in a fixation solution containing 2% glutaraldehyde and 2% paraformaldehyde in 0.1 M sodium cacodylate buffer at room temperature for 1 h and then at 4 °C for 16 h. The samples were washed, post-fixed with 1% osmium tetroxide containing 0.5% potassium dichromate and 0.5% potassium hexacyanoferrate for 1 h, washed, and stained en bloc with 2% uranyl acetate for 60 min. Following dehydration using ethanol, the tissue was infiltrated for 3 days with Epon812, oriented in silicon molds, and polymerized at 60 °C. Transverse ultra-thin 80 nm sections were cut with a UC7 ultramicrotome (Leica, Wetzlar, Germany), transferred to copper grids, and viewed under a Talos L120C transmission electron microscope (Thermo Fisher Scientific) at an accelerating voltage of 120 kV.

### 2.10. Magnetic Resonance Imaging (MRI)

For the Gd-enhanced MRI, 50 μL of Cyclolux (gadoteric acid) 0.5 mmol/mL (Sanochemia, Neufeld an de Leitha, Austria), an MRI contrast agent, was injected intravenously. MRI was performed 20 min later. Images were acquired on a 9.4T horizontal MRI system (Bruker Biospec, Ettlingen, Germany) using a cylindrical volume coil (86 mm inner diameter) for radiofrequency transmission and a 20 mm diameter surface coil for detection. During imaging, animals were anesthetized with 1–2% isoflurane supplemented with oxygen (0.7 L/min). Respiration was monitored (Small Animal Instruments, Stony Brook, New York, NY, USA), and body temperature was maintained using circulating hot water.

Gadolinium-enhanced MRI images were acquired using a 3D T_1_-weighted fast low angle shot (FLASH) sequence, with TR/TE 15/4.7 ms and pulse 15^0^, field of view 19.2 × 19.2 × 15.5 mm^3^, matrix size 192 × 192 × 31, in-plane resolution 100 µm, number of averages 4, and total scan time 6 min. 

Medical Image Processing, Analysis, and Visualization (MIPAV) software (NIH; http://mipav.cit.nih.gov, accessed on 10 February 2023) was used for data processing. Fiji ImageJ software (1.54f, National Institute of Health, Bethesda, MD, USA) and Python software (v 3.12) were used for manual segmentation of brain structural analysis and signal intensity measurements.

### 2.11. Statistical Analysis 

Statistical analyses were conducted using GraphPad Prism (v.10.2.0). Gene expression analysis employed one-way ANOVA with 5 mice per group (*n* = 5). Student’s *t*-tests were applied for Morris water maze (MWM) and open field test (OFT) data, with 5 mice per group (*n* = 5) as well. Microglial and astrocytic morphological analyses utilized 5 mice per group (*n* = 5), with 3 brain sections per mouse and 20–30 cells analyzed per section. NeuN and TUNEL analyses also employed Student’s *t*-tests, with 5 mice per group (*n* = 5). Student’s *t*-tests were used for statistical analysis of mitochondria, including the percentage of axons with mitochondria and the mitochondrial area, perimeter, and circularity. Pearson correlation coefficient assessed the linear correlation between mitochondrial radius and g-ratio. Student’s *t*-tests were applied for statistical axonal analysis, except for albumin signal intensity analysis, which also utilized Student’s *t*-tests. A significance level of *p* < 0.05 was used for all tests.

## 3. Results

After PIXE analysis confirmed an increase in cobalt levels in bodily fluids and tissues, animal behavior was assessed using the OFT and MWM. These findings were compared with the results of brain structural anatomy analysis by MRI, TEM, histological studies, immunohistochemical studies, and molecular assays. 

### 3.1. High-Dose Cobalt Toxicity Induced Reversible Systemic Illness

The acute response to high-dose cobalt was assessed over 3 days (Figure 1) by monitoring several behavioral indices of acute sickness (e.g., weight loss, lethargy, low social interaction). Within the first 24 h, mice appeared very sick, with total loss of balance and orientation, almost absent social interaction, and significant weight loss (Δ = −6.6%, *p* < 0.05). Thereafter, their condition steadily improved and reached baseline at 72 h after cobalt injection. 

### 3.2. High-Dose Cobalt Toxicity Induced Oxidative Stress and Brain Hypoxia

**Figure 2:**
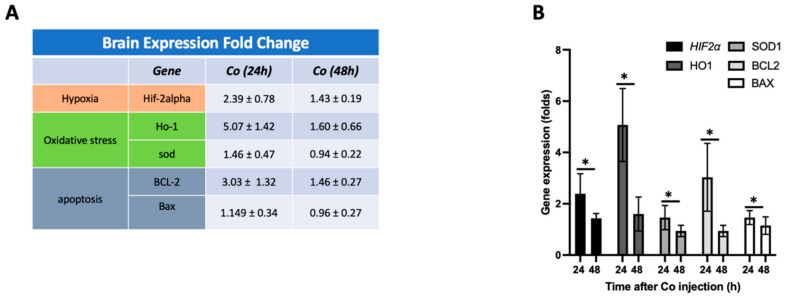
Co-injection caused an increase in the expression of genes related to hypoxia, oxidative stress, and apoptosis. (**A**,**B**) Gene expression levels of several targets, including hypoxia (Hif-2alpha), oxidative stress (Ho-1), and apoptosis (BCL-2, Bax) genes, were determined over the first 48h. The ratio of Bax to BCL-2 was determined to be 0.379 after 24 h and 0.65 after 48 h post-injection. Data expressed as percent change from baseline. Data shown are mean ± SD, * *p* < 0.05.

Brain sections were collected 24 and 48 h after high-dose cobalt injection, corresponding to the time course of the resolution of systemic illness and behavioral changes, and mRNA expression of markers of oxidative stress (*HO1, SOD1*), hypoxia (*HIF2α*), and apoptosis (*BCL2, BAX*) was analyzed. There was a notable rise in mRNA levels of HO1 and SOD1 after exposure to cobalt, reaching a peak at 24 h (HO1: 5.07 ± 1.42, *p* < 0.05; SOD1: 1.46 ± 0.47, *p* < 0.05 for both), followed by a decline at 48 h (HO1: 1.6 ± 0.66, *p* < 0.05; SOD1: 0.94 ± 0.22, *p* < 0.05 for both). The *HIF2α* mRNA level followed the same pattern (24 h: 2.39 ± 0.78, 48 h: 1.43 ± 0.19, *p* < 0.05). BCL2 and BAX did not show a statistically significant change at either timepoint (Figure 2). 

### 3.3. High-Dose Cobalt Injection Was Associated with Increased Brain Microglial and Astrocyte Activation at 24 h

**Figure 3:**
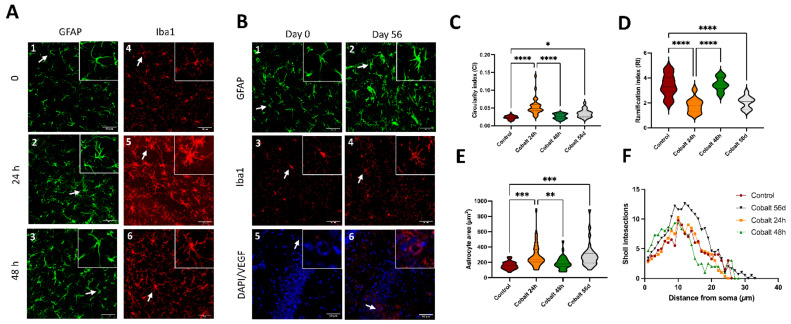
Morphometric analysis of microglial and astrocytic activation following cobalt exposure (**A**–**F**). (**A1**–**A6**) Increased immunoreactivity of Iba-positive microglia and GFAP-positive astrocytes was evident during the first 24 h after a single high-dose injection of cobalt and subsided by 48 h. (**B1**–**B4**). Low levels of Iba and GFAP immunoreactivity after 56 days of daily low-dose cobalt administration. (**C**) Swarm plot showing circularity index of the experimental groups. (**D**) Swarm plot showing ramification index of the experimental groups. (**E**) Swarm plot showing astrocytic cover area in the experimental groups. (**F**) Distinct difference between control and low-dose-cobalt-treated group (* *p* < 0.05) in Sholl intersection distribution by distance from the astrocyte’s soma. The analyses (**C**–**F**) were performed by one–way ANOVA with Tukey’s multiple comparison tests. Each group contained 5 mice (*n* = 5); for each mouse, twenty to thirty cells per region were analyzed. (**B5**,**B6**) Increased VEGF immunoreactivity, particularly localized around blood vessels, in the cobalt-treated group versus the control group; * *p* < 0.05, ** *p* < 0.01, *** *p* < 0.001, **** *p* < 0.0001.

Immunostaining for Iba-1, a microglial marker, at 24 h after high-dose cobalt injection revealed signs of activation compared to controls, with an increase in soma size and circularity index. These were less evident at 48 h (Figure 3A). Astrocyte activation, indicated by an increase in GFAP intensity, was prominent as well at 24 h and then diminished at 48 h (Figure 3A). The astrocytes exhibited signs of hypertrophy, namely an increase in the size and thickness of the processes (Figure 3E,F). 

### 3.4. Chronic Low-Dose Cobalt Injection Caused a Chronic Inflammatory Response with Increased VEGF Expression 

After 56 days of daily cobalt administration, morphological alterations suggestive of microglial activation (including increased circularity and ramification indices) and astrocyte activation (such as changes in astrocyte area and Sholl intersections) were evident in various areas of the brain, but mostly in the hippocampus and cortex, as shown in Figure 3B1–B4,C–F.

In addition, VEGF^+^ cells were greatly increased in the cobalt-injected mice after 56 days compared to control mice (Figure 3B). 

### 3.5. Chronic Low-Dose Cobalt Injection Caused Increased Cobalt Levels in Tissues by PIXE

PIXE analysis of cobalt accumulation in biological fluids and tissues after 28 days of daily IP administration yielded high levels in both blood and urine (37.24 ± 21.33 ppm and 769.42 ± 485.74 ppm, respectively), in addition to the liver (627.5 ppm) and heart (240.9 ppm), and lower levels in the brain (55.7 ppm). 

### 3.6. Chronic Low-Dose Cobalt Injection Caused Functional Behavioral Changes

**Figure 4:**
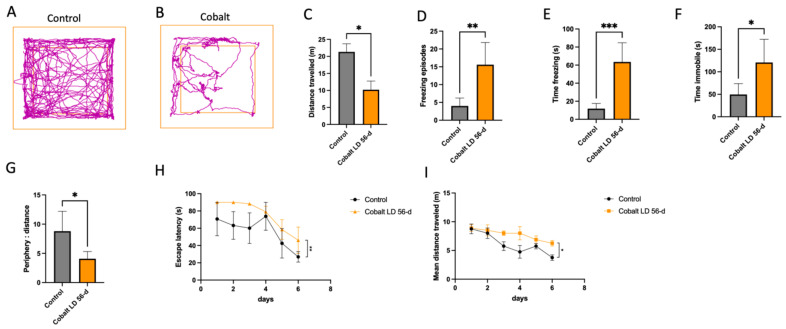
Behavioral assessment of cobalt-induced effects on locomotion and anxiety. (**A**–**G**). (**A**,**B**) Representative OFT track *plot recorded* during the 5-min test sessions (ANY-maze). The central square was designated the “central zone”, and the periphery, the “border zone”. (**C**–**G**) Locomotor activity decreased after chronic cobalt treatment, whereas anxiety-related behavior was increased. * *p* < 0.05, ** *p* < 0.01, Student *t*-test. (**H**,**I**) MWM spatial acquisition learning curves after 6 consecutive days. Data points represent mean values of escape latency of the control (black) and the cobalt-injected (orange) groups (**H**). The between-group difference was statistically significant. (**I**) Mean distance traveled in meters until island zone entry as a function of the day of training. The day of training had a significant influence on the mean distance traveled in both groups (*p* < 0.05). * *p* < 0.05, ** *p* < 0.01, *** *p* < 0.001 Student t-test. Number of mice: *n* = 5 in each group. Data shown are mean ± SEM. LD = low dose.

Functional and behavioral changes were detected in the mice injected with chronic low-dose cobalt. On the OFT, they exhibited a decreased quantity and quality of activity compared to controls. As shown in Figure 4A–G, there was a decrease in distance traveled (10.19 m vs. 21.3 m, *p* < 0.05), an increase in freezing episodes (15.6 vs. 4.0, *p* < 0.05), time spent in the frozen state (63.56 s vs. 11.82 s, *p* < 0.05), and duration of immobility (120.8 s vs. 49.5 s, *p* < 0.05), and a decrease in time spent in the center compared to the periphery (4.085 s vs. 8.791 s, *p* < 0.05). 

Thereafter, spatial learning and memory were tested with the MWM. All control mice showed the expected learning curves with improvement in learning and in locating platforms. At 56 days from injection, the cobalt-treated mice exhibited a significantly longer escape time and mean distance traveled than the control group (Figure 4H,I). 

### 3.7. Chronic Cobalt Exposure Causes Neurodegenerative Damage

#### Histology and Immunohistochemistry

**Figure 5:**
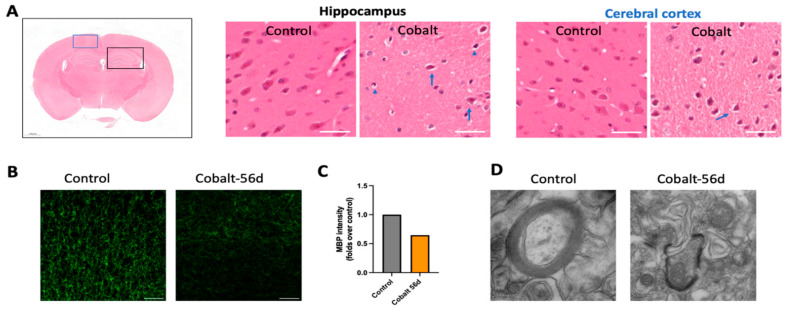
Immunohistochemistry (IHC) and ultrastructural analysis of cobalt-induced neurotoxicity. (**A**) H&E staining in the cobalt-treated group revealed small, deformed nuclei (blue arrowhead) and cracked cytoplasm (blue arrow). Other findings included hyperchromatic cells (blue arrow), cellular atrophy, shrinkage, cellular necrosis, pyknosis, and deeply stained and dark nuclei, in addition to large multipolar cells, neuronal swelling, chromatolysis, and nuclear margination. (**B**,**C**) Immunostaining with myelin basic protein (MBP) (green) in the cortex showing demyelination compared to controls with decreased staining signal intensity in the treated group; scale bar = 50 µm. (**D**) TEM analysis revealed extensive loss of myelin and axonal injury.

Macroscopic examination of the brains did not reveal any architectural disturbances. On H&E staining, neurodegeneration with nuclear disintegration and shrinking, pyknosis, and cytoplasmic cracking of the pyramidal cells were observed in the hippocampus and frontal cortex (Figure 5A). There was significant demyelination in certain areas of the brain, as shown in Figure 5B–D.

**Figure 6:**
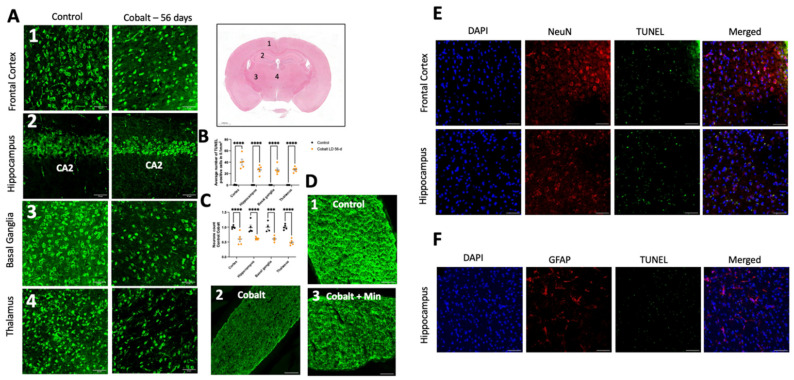
Immunostaining and quantification of neuronal loss and cell death. (**A**) Neurons were labeled with NeuN antibody in the frontal cortex, hippocampus, thalamus, and basal ganglia. (**B**) Quantification of the mean number of neurons in these brain areas indicated neuronal loss in the cobalt-treated group. (**C**) Quantification of TUNEL+ cells/area in the frontal cortex, hippocampus, thalamus, and basal ganglia. **** *p* < 0.0001, *** *p* < 0.001, Student *t*-test. Number of mice: *n* = 5 in each group. Data shown are mean ± SEM. Scale bar = 50 µm. LD = low dose. (**D1**) Illustration of normal myelin in a control mouse. (**D2**) Extensive demyelination in a cobalt-treated mouse. (**D3**) Partial preservation of myelin in a minocycline-treated mouse on MBP immunohistochemical staining. Scale bar: 50 µm. (**E**) TUNEL and neuronal marker NeuN+ staining after daily IP treatment for 56 days yielded positive findings. (**F**) GFAP and TUNEL did not overlap, indicating that astrocytes were less susceptible to cobalt toxicity.

Remarkably, the evaluation of the impact of minocycline supplementation on demyelination revealed that the group that received minocycline in addition to cobalt exhibited less demyelination on quantitative analysis than the cobalt-only group (Figure 6(D1,D2)). 

Daily cobalt treatment was associated with a notable reduction in NeuN-positive neurons, as depicted in Figure 6A,B. Subsequent analysis of brain sections revealed a significant increase in TUNEL-positive cells compared to controls, indicative of increased apoptosis (Figure 6C). Apoptotic cells, characterized by condensed and fragmented DAPI-stained nuclei, predominantly exhibited NeuN positivity (Figure 6E). Minimal co-localization with GFAP (Figure 6F) or Iba1 was observed, suggesting a neuronal origin rather than astrocytic or microglial involvement. Several variations of TUNEL signal patterns including universal, apoptotic, cytoplasmic, and dispersed were noted. The latter, primarily observed in necrosis, is marked by the breakdown of the nuclear and plasma membranes, accompanied by irregular leakage of TUNEL-positive material and cytoplasm.

### 3.8. Ultrastructural Changes Induced by Cobalt Neurotoxicity: Neuronal Damage, Axonal Degeneration, and Myelin Alterations Observed by Transmission Electron Microscopy (TEM)

**Figure 7:**
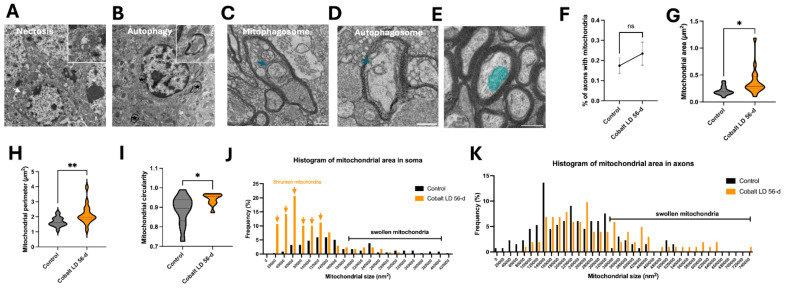
Cobalt-induced neuronal death mechanisms: necrosis, autophagy, and mitochondrial alterations. (**A**) Necrotic neuronal cell body showing the absence of a defined nuclear membrane, enlarged mitochondria (white arrow), and altered chromatin organization after 56 days of daily cobalt exposure. The inset presents a high-resolution image of the swollen mitochondria. (**B**) Autophagic activity observed in neuronal cell bodies at 56 days after cobalt treatment, with autophagosomes marked by asterisks. The inset provides a detailed view of an autophagosome. (**C**,**D**) An example of a small, degraded mitochondrion within a mitophagosome and the formation of autophagosomes (blue arrows) in an axon from a cobalt-treated mouse. Scale bar: 0.5 µm. (**E**) Cross-sectional view of a myelinated axon with mitochondria (blue) in a control mouse; scale bar: 0.5 µm. (**F**) Percentage comparison of myelinated axons containing mitochondria between cobalt-exposed and control mice. (**G**–**I**) Cross-sectional analysis of mitochondrial size (**G**), perimeter (**H**), and circularity (**I**) in axons. Violin plots show the full distribution of measurements. * *p* < 0.05, ** *p* < 0.01, NS (not significant); Student’s *t*-test. A total of 300 mitochondria were analyzed in each group, with data expressed as mean ± SEM. (**J**,**K**) Frequency distribution of mitochondrial area in neuronal cell bodies (**J**) and axons (**K**) between control and cobalt-exposed groups following two months of daily treatment. Arrows indicate a higher occurrence of shrunken mitochondria. Number of mitochondria analyzed: *n* = 300 per group.

TEM revealed several forms of neuronal damage in the same brain regions in the cobalt-treated group. The neuronal somas showed loss of a distinct nuclear membrane or total loss of the nuclear envelope, and the mitochondria and other organelles were enlarged and swollen (Figure 7A), indicating different degrees of necrosis. Autophagic neurons were seen with autophagosomes, which appeared as double-membrane-bound autophagic vesicles in somas (Figure 7B). Morphological analysis, performed as previously described [30], demonstrated a higher occurrence of smaller mitochondrial areas in the somas of neurons from the treated group compared to controls, indicating mitochondrial shrinkage (Figure 7J). Conversely, analysis of the mitochondrial area in axons revealed a greater incidence of swelling in the treated group (Figure 7K).

**Figure 8:**
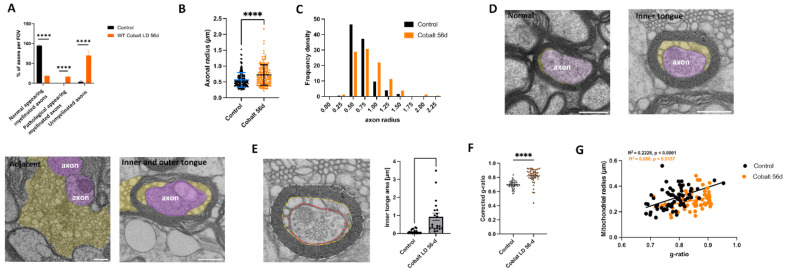
Phenotypic quantification of optic nerve alterations following cobalt treatment. (**A**) Analysis was performed on optic nerve cross-sections on a total area of >320 µm^2^ with >200 axons per group; all the axons in the field of view were counted. **** *p <* 0.001, unpaired *t*-test. (**B**) Plot showing increased axonal radius in the cobalt-treated group compared to controls. **** *p* < 0.0001, unpaired *t*-test. (**C**) Histogram showing axonal radius density. (**D**) Electron micrograph of optic nerves showing myelin pathology. Membrane tubules (orange) emerge at the inner tongue of the cobalt-treated mice. Occasionally, tubulations (purple) are seen at the outer tongue of myelinated axons associated with tubulations at the inner tongue. At places where most compact myelin is lost, membrane tubules loop out and leave partially demyelinated axons behind. Tubules could also be found next to demyelinated axons. (**E**) Illustration of corrected g-ratio measurement. Three lines are drawn for the area measurement: the outline of the fiber (stippled white line), the outline of the inner border of the compact myelin (orange), and the axon (red). Scale bar = 500 nm. The inner tongue area was increased in the low-dose-cobalt-treated group compared to the control. (**F**) Corrected g-ratio measurements reveal a progressive decrease in compact myelin in the cobalt-treated group compared to controls. Number of mice: *n* = 5, >20 axons each. **** *p* < 0.0001, unpaired *t*-test. (**G**) Scatter plot illustrating the relationship between the mitochondrial radius and the g-ratio. Pearson’s *r* test.

Varying forms and degrees of optic nerve axonal degeneration due to cobalt neurotoxicity were noted (Figure 8A). Axons had non-uniform sizes and shapes, and there were fewer axons in tissues from the cobalt-treated mice than controls. The surrounding myelin in most axons appeared fractured, while mitochondria and other organelles exhibited swelling or a dark, dense appearance (Figure 5D). Autophagosomes and mitophagosomes were present within the axons, and neurites displayed abnormal enlargement and swelling (Figure 7C,D). These findings collectively indicate dystrophy, suggesting severe cell death and/or axonal degeneration. Quantifying the axon diameter yielded a significantly higher value in the cobalt-treated than in the control group (Figure 8B,C), supporting axonal swelling and degeneration. 

To further investigate the conspicuous axonal structural change due to cobalt toxicity, the phenotype of the cobalt-treated group was assessed. Owing to its significant level of myelination and consistent pattern, the optic nerve was subjected to detailed ultrastructural analysis using methods similar to those of Mesckhat et al. [31] Significantly widespread axonal demyelination was observed at 56 days after cobalt injection, in addition to the emergence of membrane processes resembling the *shiverer* mouse model phenotype, particularly at the myelin inner tongue or both the inner and outer tongue (Figure 8D,E). Membrane tubules could be seen adjacent to non-myelinated axons (Figure 8D). Quantitative analysis of myelin tubulation revealed that approximately 60% of the axons were demyelinated (Figure 8A). The process of compact myelin loss included a transitional phase marked by the emergence of membrane tubules and resulted in pathological-appearing myelinated axons (Figure 8A).

To assess the reduction in thickness of the compact myelin sheath, both the area occupied by the myelinated fiber and the area encompassed by the tubulated inner tongue and the axon were investigated (Figure 8E). The thickness of the remaining compact myelin, termed the corrected g-ratio, was measured by dividing the calculated axonal diameter by the calculated myelinated fiber diameter, with the exclusion of the inner tongue area [31]. As shown in Figure 8F, there was an increase in the g-ratio after 56 days of cobalt treatment. The presence of any potential changes in energy provision by mitochondria was evaluated by alterations in mitochondria within the myelinated axons [32,33,34]. The percentage of cross-sectional myelinated axons with at least one mitochondrion did not differ between the cobalt-treated and control groups (Figure 7F). The axonal mitochondria in the cobalt-treated group had a higher cross-sectional area, perimeter, and circularity (Figure 7G–I). However, there was no correlation between the mitochondrial radius and the g-ratio (Figure 8G), demonstrating a failure of homeostatic compensation. 

### 3.9. Chronic Low-Dose Cobalt Administration Induced Hypoxia with Disruptive Effects 

**Figure 9:**
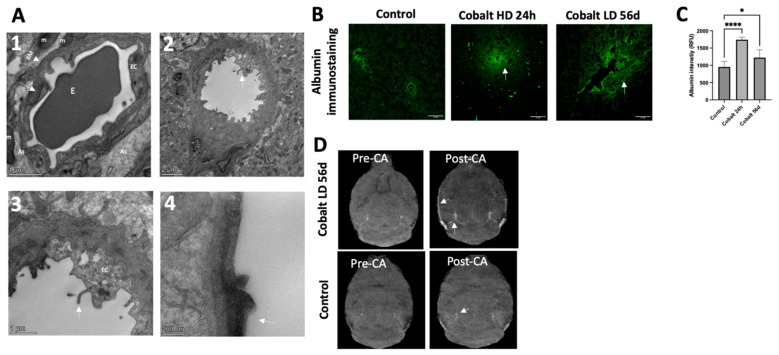
Cobalt-induced disruption of blood–brain barrier (BBB) integrity and enhanced vascular permeability. (**A1**) Capillary from the cerebral cortex of a control mouse (high magnification). Note the single layer of endothelial cells surrounded by a layer of basement membrane, forming an intact BBB. An erythrocyte can be observed in the lumen of the capillary. (**A2**,**A3**) Numerous vesicles are present in the cytoplasm of an endothelial cell from the cortex and hippocampus of a cobalt-treated mouse. Microvilli and their fragments (white arrows) are present in the capillary lumen. (**A4**) The tight junctions were unclear, and basal laminas were partially collapsed. (**B**,**C**) Immunohistochemical analysis revealed the presence of albumin within brain tissues. In control mice, albumin immunostaining distinctly showed localization within blood vessels, but in cobalt-treated mice, albumin extravasation (green, indicated by arrows) was observed. Number of mice: *n* = 5 each. One-way ANOVA. * *p* < 0.05, **** *p* < 0.0001. (**D**) Cross-view images acquired before (Pre-CA) and after (Post-CA) injection showing increased signal intensity (white arrow), indicating BBB leakage. CA = contrast agent.

Blood–brain barrier (BBB) integrity was evaluated by MRI which showed increased contrast agent signal intensity (Figure 9D). In the control group, TEM imaging demonstrated normal structural integrity of the capillaries (Figure 9(A1)). There was a single layer of endothelial cells surrounded by a layer of basement membrane and continuous basal laminas with an intact tight junction. In the cobalt-treated group, structural abnormalities were evident in the capillary endothelia, and numerous flask-shaped invaginations were present on the luminal surface of the endothelial cells (Figure 9(A2,A3)). Although occasionally the tight junctions were unclear and the basal laminas partially collapsed, there were no evident gaps between tight junctions. Immunohistochemical staining for albumin demonstrated albumin extravasation in the cobalt-treated group compared to the control group in which albumin staining could be clearly seen within the blood vessel (Figure 9B,C). These results indicate that cobalt toxicity can cause BBB leakage.

## 4. Discussion

This study investigated the physiologic effect of increased cobalt levels in blood, urine, and tissues in a mouse model. Hypoxic brain damage with BBB leakage was observed. The neurotoxicity after acute exposure was reversible, but chronic exposure led to functional damage. The structure–function correlation was analyzed. 

Cobalt neurotoxicity has well-established signs and symptoms, ranging from minor mood variations to cognitive and memory deficits, sensorineural deafness, and optic atrophy, among others [1,6,35,36]. For purposes of this study, we created two mouse models that simulated acute high-dose and chronic low-dose cobalt toxicity. In agreement with other studies [37,38,39], we showed that following a single IP injection, cobalt excretion occurs rapidly, with most of the administered dose eliminated within 2 h. However, following repeated parenteral injections, cobalt tends to accumulate in tissues in a dose- and time-dependent manner, with variations among the different organs. As hypoxia is induced, organs dependent on high mitochondrial energy, such as the brain and optic nerves, are easily affected. It is noteworthy that we found the highest levels of cobalt deposition in the liver and heart, and to a lower extent in the brain. The accumulation in the liver and kidneys may be attributable to the involvement of these organs in detoxification, given that cobalt is mainly excreted through urine and less through bile and feces [39]. In a study in rabbits, Apostoli et al. reported that cobalt tended to accumulate in the brain after 1 month of continuous IV infusion, with the rabbits exhibiting severe organ damage and eye and auditory system impairment on histologic evaluation [40]. In another study in rats, cobalt induced only subtle or moderate neurotoxicity after daily IP administration, although it accumulated significantly in the brain [10]. To our knowledge, there are no studies of cobalt concentration in the brains of patients with cobalt toxicity. 

Cobalt-induced toxicity has been well documented following local and systemic exposure to cobalt-containing metal-on-metal implants in patients after hip arthroplasty [41]. The cobalt originating from the alloy implants stimulated macrophage activation, initiating an immune response that led to the secretion of various proinflammatory mediators, such as tumor necrosis factor alpha (TNF-a), interleukin (IL)-1beta (IL-1β), IL-6, and IL-8 [42,43]. These findings were supported by several in vitro studies demonstrating an increased production of these cytokines in different cell lines exposed to cobalt [44,45]. In our mouse model, morphological changes in microglia (increased Iba1 immunoreactivity associated with increased circularity index, soma size, and Sholl’s intersections) were evident in various brain regions, including the cortex, hippocampus, basal ganglia, and thalamus. Similarly, there was increased activity of astrocytes (increased GFAP immunoreactivity) in the hippocampus. These changes were apparent 24 h after acute cobalt toxicity and seemed to subside at 48 h, coinciding with a decrease in cobalt concentration within the mouse body. 

A single acute high dose of cobalt induced functional and behavioral deterioration characterized by systemic illness and weight loss. Weight loss has been previously reported among the signs of cobalt toxicity [41]. These findings all subsided after 48 h, together with the elimination of the cobalt from the body. By contrast, chronic cobalt intoxication for almost 2 months caused significant behavioral disturbances. Mice were weak, with a marked difference in OFT results compared with controls. They showed decreased exploratory behavior and locomotor activity and increased anxiety-like behaviors. Accordingly, previous studies in patients with cobalt toxicity reported depressed mood, development of psychotic illnesses, and anxiety [35,36] in addition to impaired memory [36]. We also observed notable between-group differences in the MWM test. The cobalt-treated mice exhibited significantly increased escape latency times and mean distances traveled, suggesting a deterioration in cognitive function (spatial learning) and memory retrieval. In both the hippocampus and cortex, H&E staining revealed a distinct rise in cellular degeneration, characterized by nuclear disintegration, cytoplasmic cracking, and pyknosis. The in vivo TUNEL assay supported apoptosis, mostly affecting NeuN-positive cells. These observations strongly suggest neuronal death due to cobalt toxicity [46,47].

Using TEM, we found that cobalt toxicity caused axonal injury with a clear pattern of demyelination, including loss of uniformity in size and shape compared to controls. We incorporated the optic nerves in our investigation to gain deeper insight into the observed alterations. Cobalt toxicity led to a transformation from a fully myelinated nerve into a *shiverer*-like phenotype, characterized by a deficiency or absence of compact myelin, abnormal myelin structure, and higher g-ratio [32,48,49,50]. In response to the diminished myelin density, the myelinated axons exhibited hypertrophy. While there was an overall increase in mitochondrial size parameters, they did not correlate with the g-ratio of the axon, suggesting a lack of homeostatic compensation of the mitochondria to the increased energy demand caused by demyelination [51]. Consequently, these ultrastructural adaptations in myelinated axons may serve as indicators of the pathological changes associated with cobalt toxicity. In addition, several forms of neuronal damage and death were seen on TEM. These findings suggest that chronic cobalt toxicity induces a range of cellular damage characterized by distinct types of cell death. Necrosis emerged as a prominent feature, marked by cell swelling, loss of membrane integrity, and subsequent inflammatory responses. In contrast, autophagy served as a potential survival mechanism, with cells attempting to manage stress by degrading damaged components, including the formation of autophagosomes to engulf cellular debris and mitophagosomes targeting dysfunctional mitochondria for degradation. Thus, while necrosis indicates severe damage and irreversible cell death, autophagy reflects a compensatory response that can promote cell survival or lead to programmed cell death if the stress persists.

According to previous experimental studies, metal toxicity compromises BBB integrity through various mechanisms, including oxidative stress, inflammation, and disruption of tight junctions between endothelial cells [52,53,54,55,56]. The exact mechanisms underlying the resultant elevation in vascular permeability in vivo remain unclear. In our mouse model, cobalt toxicity disrupted BBB integrity, as indicated by the increase in signal intensity of the contrast agent on MRI. This was supported by our observation of extravasation of albumin, with a marked difference from the control group. CoCl_2_ is known to be associated with hypoxia [57]. Our real-time PCR results showed increased mRNA levels of *HIF2α* in the cobalt-injected group, in addition to in *HO1* and *SOD1*. These changes were evident 24 h after acute cobalt exposure but appeared to diminish by 48 h, coinciding with a decrease in cobalt concentration in the mice. This pattern was similar to the transient changes observed in GFAP and Iba-1 expression, which also correlated with the temporary increase in cobalt toxicity as mentioned before. These findings were accompanied by an elevated expression of VEGF, often associated with chronic ischemia. VEGF gene expression is known to be upregulated in response to hypoxia, highlighting its role as a molecular mediator in the adaptive response to reduced oxygen levels [58]. Originally, however, it was described as a vascular permeability factor. VEGF expression may link hypoxia and vascular leakage in the central nervous system in vivo [59]. Recent studies revealed that ischemia increases the number of vesicles in the cytoplasm of brain vascular endothelial cells which transport molecules across the BBB into the brain and are correlated with the severity of BBB disruption [60,61,62]. In the present study, the number of endothelial vesicles was increased in the cobalt-injected group. In instances of significant endothelial damage, the loss of cytoplasmic integrity could potentially contribute to BBB disruption [60,63]. The endothelial cells within ischemic regions frequently exhibited qualitative signs of distress (edema, microvilli projection to the lumen); nevertheless, there was no indication of endothelial cell necrosis or apoptosis. Hence, the observed rise in BBB permeability does not seem to be directly linked to a pronounced loss of endothelial integrity. This discrepancy may stem from the milder ischemia caused by cobalt in our study compared to previous research involving severe endothelial damage [60,64].

Earlier studies leveraging TEM to evaluate tight junctions have often been considered to support the role of tight junctions in BBB permeability [65]. They typically revealed discrepancies such as gaps or widening between endothelial cell membranes at the tight junction complex. However, this approach has faced criticism for its subjective nature [66]. In the present study, although some tight junctions exhibited indistinct features, there was no clear evidence of gaps or expansions between endothelial cell membranes. 

The concurrent administration of minocycline with cobalt in mice had a partial neuroprotective effect, characterized by a reduction in demyelination and preservation of myelin integrity. Minocycline, an anti-inflammatory tetracycline derivative that modulates macrophage activity, inhibits matrix metalloproteinases (MMPs) and inflammation, demonstrating neuroprotective potential by lowering MMP activity in response to microvascular damage [67,68,69,70,71]. In our study, cobalt chloride, a hypoxia-inducing factor, triggered neurotoxicity through inflammatory activation of microglia and macrophages, leading to secondary damage such as mitochondrial impairment and ischemic-like effects. By administering minocycline, we aimed to reduce this inflammatory response and alleviate ischemia-related damage. As noted in the introduction, recent studies indicate minocycline’s role in reducing hypoxia-induced microglial excitation and alleviating neonatal hypoxic insults on brain organoids [26,27]. Our findings align with prior research showing minocycline’s capacity to reduce microglial activation, thereby improving motor function during stroke recovery. This observed neuroprotective effect positions minocycline as a promising agent for clinical interventions targeting neuroinflammatory processes associated with ischemic pathology.

In conclusion, the present study demonstrates that chronic cobalt exposure in mice induces hypoxic damage to brain tissue, leading to functional behavioral changes in correlation with molecular and histological findings. The neurodegeneration is characterized by the activation of microglia and astrocytes and demyelination, as well as endothelial dysfunction and BBB damage with increased permeability and leakage. By elucidating the intricate interplay between cobalt toxicity and neurodegenerative processes, our study contributes to the growing body of knowledge aimed at addressing the neurological consequences of heavy metal exposure. 

Additionally, while we observed a partial neuroprotective effect of minocycline, resulting in some preservation of myelin likely due to its modulation of inflammatory cell activity, further research is needed to fully elucidate the mechanisms underlying minocycline’s action in the context of cobalt-induced neurotoxicity and hypoxia.

## Figures and Tables

**Table 1:** List of primers.

ACTB_F	TAG GCA CCA GGG TGT GAT GGT
ACTB_R	CAT GTC GTC CCA GTT GGT AAC A
HIF2α_F	CAA CCT GCA GCC TCA GTG TAT C
HIF2α_R	GTG GCT TGA ACA GGG ATT CG
HO1_F	CAG GG TCC AGA GAA GGC T
HO1_R	TCT TCC AGG GCC GTG TAG AT
SOD1_F	GCC CGG CGG ATG AAG A
SOD1_R	CGT CCT TTC CAG CAG TCA CA
BCL2_F	CCT GTG GAT GAC TGA GTA CCT
BCL2_R	GAG CAG GGT CTT CAG AGA CA
BAX_F	CTG AGC TGA CCT TGG AGC
BAX_R	GAC TCC AGC CAC AAA GAT G

## Data Availability

The data that support the findings of this study are available from the corresponding author upon reasonable request.
